# A case report and literature review on positive outcomes achieved through radiotherapy in a patient with composite hemangioendothelioma

**DOI:** 10.3389/fonc.2025.1656872

**Published:** 2025-08-29

**Authors:** Fei Zhang, Cha Luo, Ying Zeng, Zhonglian Wang, Hongting Jiang, Qing Ye, Wei Jian, Jing Zhang, Qiaofen Fu

**Affiliations:** Department of Radiation Oncology, The First Affiliated Hospital of Kunming Medical University, Kunming, Yunnan, China

**Keywords:** composite hemangioendothelioma, CHE, radiation, radiation therapy, vascular neoplasm

## Abstract

**Background:**

Composite hemangioendothelioma (CHE) is a rare borderline vascular neoplasm, typically characterized by a heterogeneous composition of benign, intermediate, and malignant vascular elements. Metastasis occurs occasionally, yet local recurrence remains relatively common. CHE frequently manifest in the deep dermis and subcutaneous soft tissues of the distal limbs, and are rarely observed in bone or vertebral column. We present a case that originally occurred in the lumbar spine.

**Case presentation:**

A 75-year-old male patient presented with progressively worsening pain in the lumbar and gluteal regions, accompanied by impaired mobility. Lumbar spine MRI and PET-CT imaging revealed abnormal signals in the left side of the L5 vertebral body and the left pedicle, indicative of potentially aggressive pathological changes. Pathological biopsy under general anesthesia confirmed a diagnosis of CHE. Given the impossibility of completely resecting the lesion, the patient underwent radiation therapy. After a course of radiotherapy (60Gy/30 fractions), the patient was discharged from the hospital. The patient has been followed up for a period of 20 months with no evidence of recurrence.

**Conclusion:**

CHE refers to a rare type of tumor that rarely involves the spine. We present a case of CHE occurring in the L5 vertebral body. Given the lack of definitive indications for surgical intervention, radiation therapy was administered, resulting in highly satisfactory therapeutic outcomes and an excellent prognosis.

## Introduction

Composite hemangioendothelioma (CHE) is extremely rare in clinical practice. Currently, there are no exact incidence statistics available (1, 2). CHE characterized by features that lie on a spectrum between benign and malignant (1). It was initially reported by Nayler et al (1, 2). Composite hemangioendothelioma mainly consists of vascular tissues of different grades, including benign, intermediate, and low-grade malignant vascular components (3). In a systematic review study by Reljic, M. et al, a total of 105 CHE cases were included, with only 2 deaths reported, yielding a mortality rate of 1.90% (4). Currently, the number of reported CHE-related deaths in the literature is extremely low (4). Given the limited sample sizes in relevant studies, the data may have certain limitations. Deaths in patients with composite hemangioendothelioma are primarily attributable to relentless local tumor progression rather than distant dissemination (5). While the mortality rate associated with this disease remains relatively low, the local recurrence rate is notably substantial (6). This tumor exhibits a degree of aggressiveness, however, it rarely metastasizes (7). The reported local recurrence rates vary across studies, all consistently indicate that the risk remains high even after radical surgical resection (4). Infiltrative growth and satellite nodules contribute to the high local recurrence rate (8).

To date, there are no established treatment guidelines for CHE (7). Complete surgical resection remains the standard treatment method for CHE, and it has been associated with excellent outcomes (8). However, the prognosis and treatment for patients with tumors that cannot be completely resected remain uncertain. Here, we present a case of a patient with typical lumbar CHE diagnosed through pathological biopsy. Considering the infeasibility of complete resection of the lesion, the patient underwent radiation therapy and achieved a favorable prognosis.

## Case presentation

A 75-year-old male patient presented to our hospital in June 2023 with a two-month history of progressively worsening pain in the lumbar and gluteal regions, necessitating the use of a crutch for slow ambulation over the past two weeks. These symptoms were not associated with physical activity and did not subside following periods of rest. There were signs of tenderness and percussion pain in the L5 vertebral body, and the patient had no history of trauma. Neurological examination indicated difficulty in lifting feet of both lower limbs, and the proximal muscle strength of the lower limbs was grade 5, and the distal muscle strength was grade 3.

**Figure 1:**
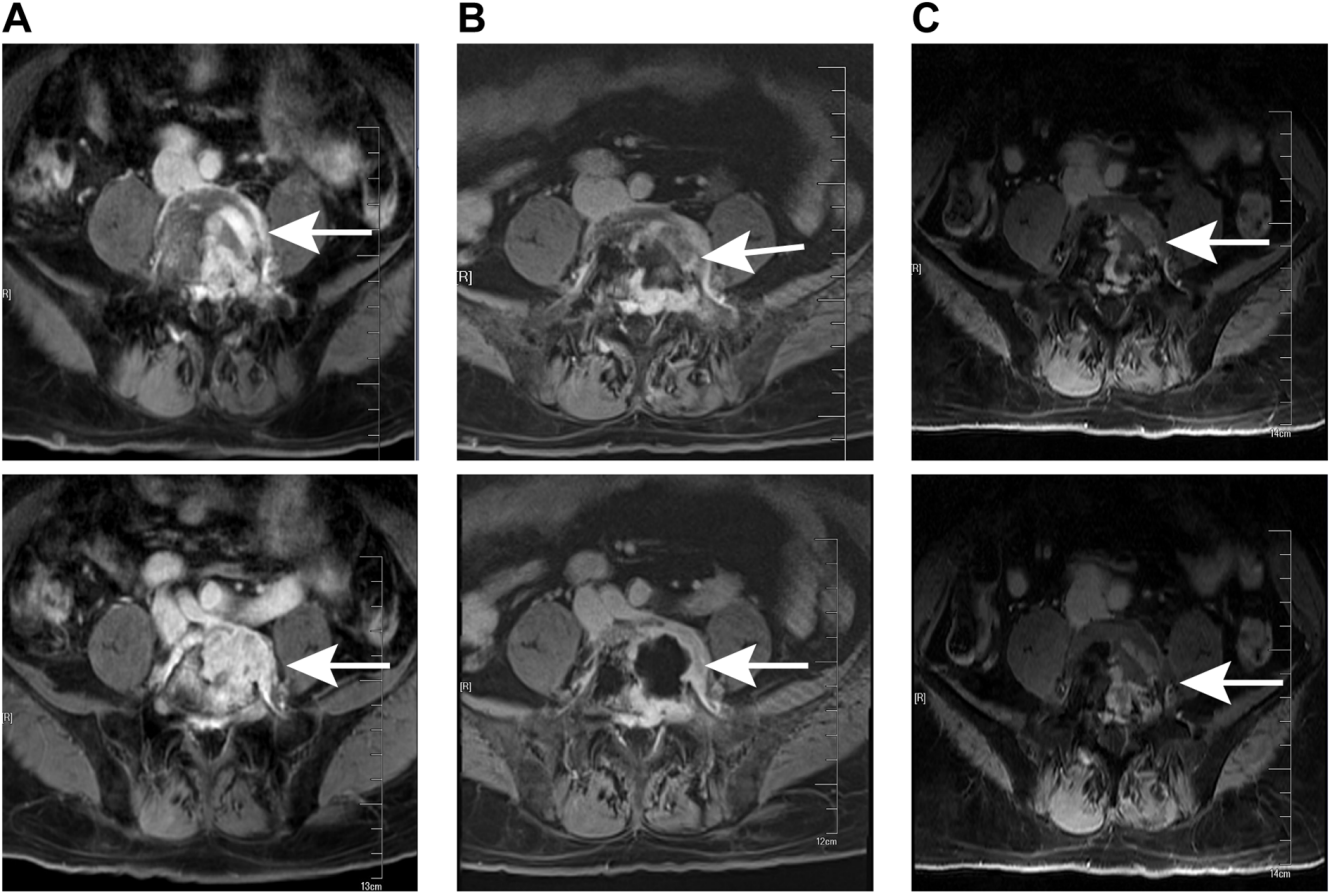
MRI examination at different stages of treatment. **(A)** Preoperative lumbar MRI showed a lesion in the L5 (white arrows). **(B)** MRI conducted prior to radiotherapy following the surgical operation revealed the lesion in the L5 (white arrows). **(C)** The latest follow-up MRI examination indicated that there was no recurrence of the L5 pyramidal lesion (white arrows).

**Figure 2:**
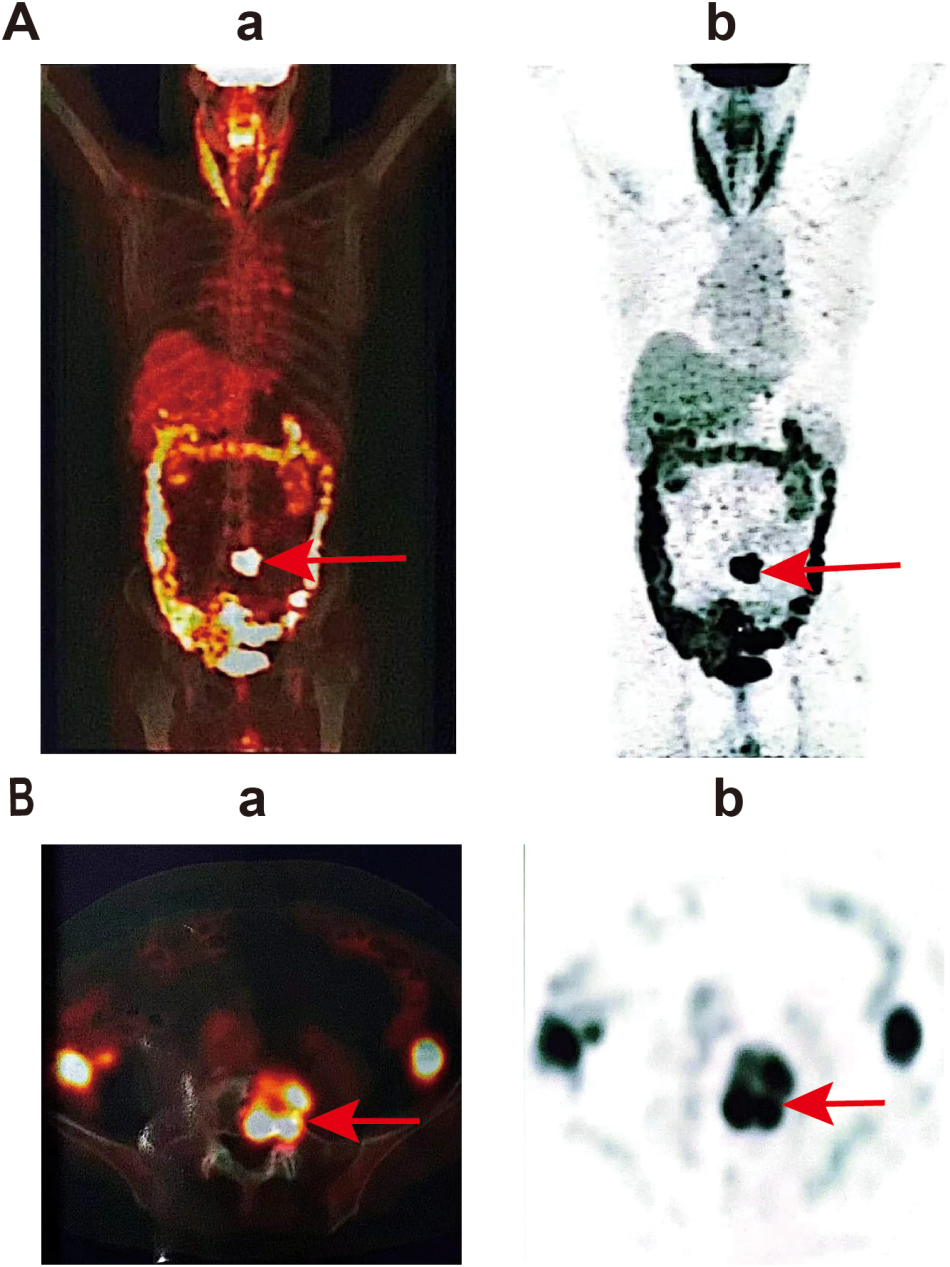
PET-CT examination showed the lesion in L5. (a) Positron emission tomography image, (b) computed tomography image. **(A)** represents a whole-body scan image, showing the overall metabolic activity of the patient; **(B)** is a local scan image, which is a transverse view, and the area pointed to by the red arrow shows the high metabolic activity in the local region.

Lumbar MRI examination revealed abnormal signal intensity with T1 prolongation and T2 shortening in the left side of the L5 vertebral body and the left pedicle, accompanied by a protrusion toward the left posterior region. The anterior and superior margins of the vertebra exhibited irregular contours, and contrast-enhanced imaging demonstrated heterogeneous enhancement, suggestive of invasive changes, raising suspicion for tumor involvement (**Figure 1A**). Subsequent PET-CT examination identified osteolytic bone destruction with irregular borders, areas of sclerosis, and soft tissue mass formation in the corresponding region. Following intravenous administration of the tracer 18F-FDG, there was increased radiotracer uptake, with a maximum standard uptake value (SUV) value of 10.7, further supporting the likelihood of a neoplastic process (**Figure 2**). The blood test results showed no significant abnormalities.

**Figure 3:**
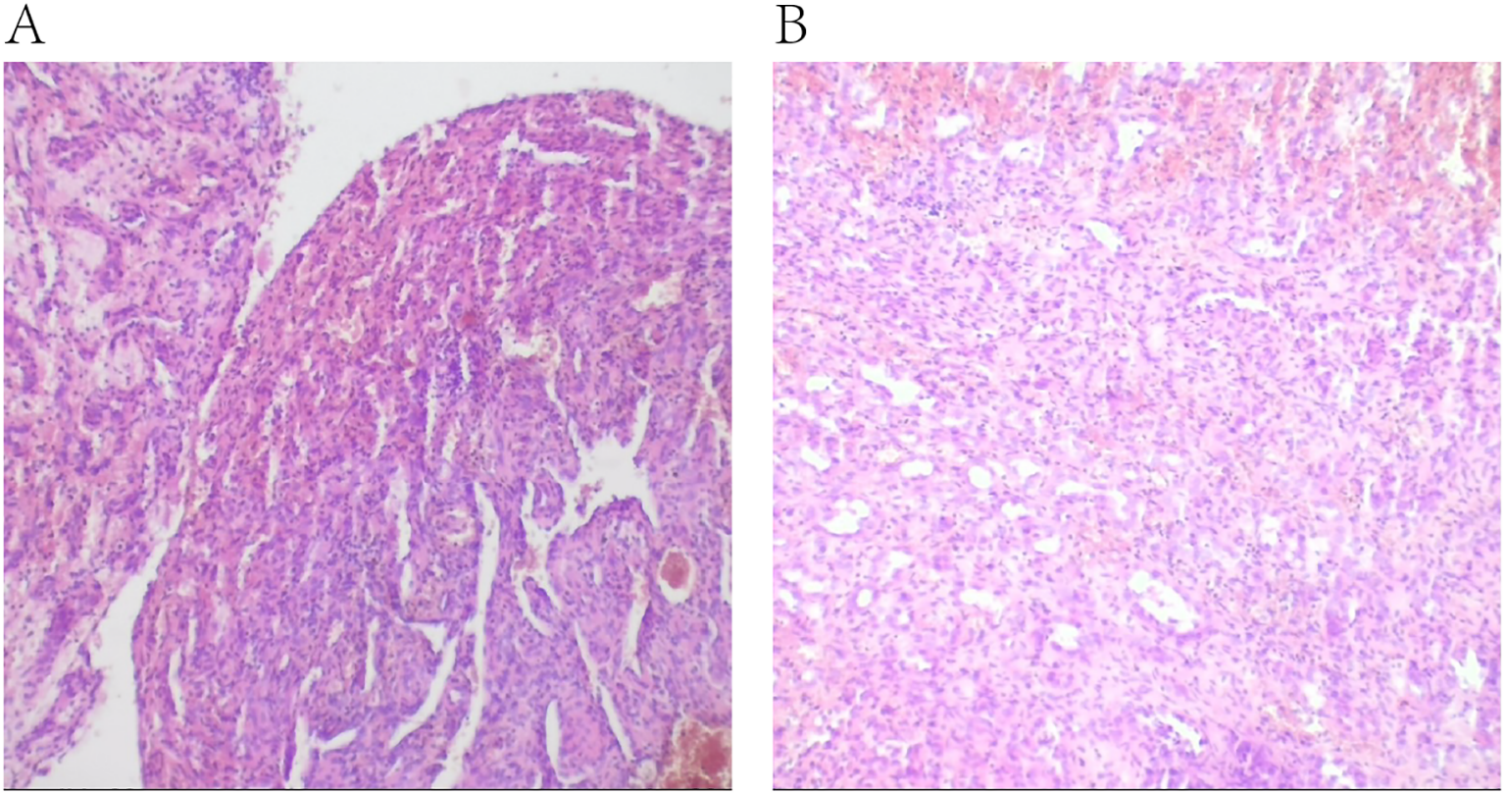
Histopathological examination after hematoxylin and eosin staining of the surgical specimen. **(A, B)** were taken from the same tissue specimen, showing pathological features in different fields of view.

Following the completion of imaging and hematology tests, a percutaneous L5 vertebroplasty using polymethyl methacrylate (PMMA) bone cement was performed to prevent a potential compression fracture. Furthermore, a biopsy was conducted for further diagnostic evaluation. To determine the pathological type of the patient’s tumor, hematoxylin and eosin (HE) staining of the biopsy specimen was conducted, along with immunohistochemistry analysis for commonly recognized tumor markers. Immunohistochemistry showed the positive of CD34, Ki-67. However, TP53, Cytokeratin (CK), Epithelial Membrane Antigen (EMA), PR, Myelin Basic Protein (MBP), CD34, GFAP and Leu-7, HNK-1, CD57, S100 protein, Neuron-Specific Enolase (NSE), SOX10, Oligodendrocyte Transcription Factors 2 (Olig-2), CD56, Synaptophysin (SYN), Smooth Muscle Actin (SMA), Vimentin were negative. The diagnosis of composite hemangioendothelioma was confirmed through a combination of HE morphology and immunohistochemistry results, along with joint consultations from pathologists in our hospital and pathologists from Ruijin Hospital, affiliated with Shanghai Jiao Tong University School of Medicine (**Figure 3**).

After the surgical procedure, the patient continued to experience significant pain in the left buttock, precluding independent ambulation. The Visual Analogue Scale (VAS) pain score was recorded at 8 points. Following administration of 20 mg oxycodone sustained-release tablets every 12 hours, there was a slight alleviation of pain symptoms, with the VAS pain score decreasing to 4 points. Ambulation remained possible only with reliance on a wheelchair or crutches for slow movement. A follow-up lumbar MRI revealed that the L5 vertebral body was filled with bone cement and exhibited ring enhancement around it (**Figure 1B**). After multi-disciplinary consultation involving medical oncologists, radiation oncologists, orthopedic surgeons, neurologists, neurosurgeons, imaging specialists, and pain management physicians, local radiotherapy was recommended. Consequently, the patient was referred to the radiotherapy department for treatment. Following completion of positioning and CT scanning, a VMAT plan (60 Gy/30 fractions) was devised under conditions ensuring spinal cord safety. Concurrently, 125 ml of mannitol was administered intravenously daily post-radiotherapy to mitigate spinal cord edema. After receiving 12 Gy/6 fractions of irradiation, the patient’s pain was markedly relieved, with the VAS pain score reducing to 5 points without oral oxycodone sustained-release tablets. The dose of oxycodone sustained-release tablets was subsequently reduced to 10 mg per dose, administered every 12 hours. Following 20 Gy/10 fractions of irradiation, the pain was further significantly alleviated, with the VAS pain score decreasing to 2 points without oral oxycodone sustained-release tablets, enabling the patient to ambulate slowly without crutches. The remaining 40 Gy/20 fractions were continued during the course of radiotherapy. During this period, the patient experienced occasional foot numbness and five episodes of diarrhea, which were effectively managed with antidiarrheal treatment using montmorillonite powder and loperamide. Upon completion of radiotherapy, the patient experienced complete resolution of pain, and normal ambulation was successfully restored. Subsequently, the patient was enrolled in the follow-up phase.

Follow-up evaluations were conducted at 3-month intervals. During each follow-up, the patient underwent a clinical examination, quality-of-life assessment, and lumbar MRI. The lumbar MRI performed 3 months after radiotherapy revealed no abnormal signals in the left portion of the L5 vertebral body or the left pedicle, indicating complete resolution of the lesion (**Figure 1C**). As of the latest follow-up, there has been no evidence of disease progression or tumor recurrence for over 20 months, and the patient reported no recurrence of pain. Ongoing follow-up will be maintained.

## Literature review

After reviewing the literature, a total of 13 similar cases were listed (**Table 1**), of which only 3 occurred in the bones, with 1 case in the cervical vertebra, and the others in soft tissues such as skin, limbs, brain, and spleen. Almost all of these cases were treated solely with surgery. Only 3 cases underwent adjuvant radiotherapy after surgery; one case was reported by Nakamura et al, another was reported by Sakamoto A et al, and the other was a recurrent intracranial MVIH patient who had a 3×3 cm enhancing extraaxial lesion in the left CPA and middle cranial fossa in 1985 and underwent subtotal resection of the intracranial lesion. Postoperative adjuvant radiotherapy was administered due to residual tumor. The tumor recurred after 9 years, and a second operation was performed, and the patient received additional radiotherapy (the dose is unknown). The tumor remained stable 2.75 years after the operation.

**Table 1:** Details of clinical and treatment in patients with CHE reported in the English literature.

Author	Case, year	Gender, Age	Clinical manifestation	Tumor location	Histopathological classification	Treatment	Follow-up period(month)	Outcome (Recurrence or not)
Avellino, A. et al. (9)	1 1999	75/F	Hearing loss, Vertigo, Vomiting, Loss of taste	Brain	Masson	Surgery, Adjuvant radiotherapy (50Gy/25Fr)	33	Yes*
Utaş, S. et al. (10)	2 2008	62/F	Asymptomatic	Left forearm	CHE	Interferon	NA	No
Tsai, J.-W. et al. (11)	3 2011	18/F	Asymptomatic	Sole of the foot	CHE	Surgery	7	No
	2011	15/F	Pharyngeal foreign body sensation	Hypopharynx	CHE	Surgery	18	No
	5 2011	49/F	Dysphonia, Dyspnea	Nasal cavity	CHE	Surgery	15	No
	6 2011	8/M	Asymptomatic	Skin	CHE	Surgery	15	No
Karaman, B. et al. (12)	7 2012	56/M	Right upper quadrant pain	Multiple hepatic masses	CHE	Yttrium-90	12	No
	8 2012	46/F	Asymptomatic	Neck mass	CHE	Surgery	NA	No
Liau, J.-Y. et al. (13)	9 2013	24/F	Hair loss	Painful firm nodules on the temporo-occipital scalp	CHE	Surgery	12	No
Sakamoto A et al. (14)	10 2017	40/F	Mass on the leg and sole	leg and sole	CHE	Surgery, Adjuvant radiotherapy (NA)	30	No
Nakamura, S. et al. (7)	11 2022	80/F	Right upper limb paralysis, Numbness in all four limbs, Progressive motor dysfunction	Cervical spine	CHE	Surgery, Adjuvant radiotherapy (50 Gy/25 Fr)	21	No
Deng, Y. et al. (8)	12 2023	21/F	Leg pain, Limited mobility	Pubis	CHE	Surgery	60	No
	13 2023	60/M	Hip joint pain	Ilium	CHE	Surgery	NA	No
Our case	14 2023	75/M	worsening pain in the lumbar and gluteal regions, impaired mobility	lumbar spine.	CHE	Surgery, Definitive radiotherapy (60Gy/30Fr)	20	No

*: In 1985, the patient's brain CT and MRI scans revealed a 3×3 cm enhancing extraaxial lesion in the left cerebellopontine angle and middle cranial fossa, with destruction of the petrous pyramid and extension into the jugular foramen and the posterior aspect of the middle ear. The patient underwent a left suboccipital craniectomy for subtotal resection of the lesion. Postoperative radiotherapy was administered to the left posterior fossa with a total dose of 45 Gy. Nine years after the surgery, MRI indicated a recurrence. After the recurrent surgery, the patient received additional radiotherapy (the dose is unknown).

NA: Not Applicable.

## Discussion

Vascular endothelioma is a type of tumor originating from the vascular endothelium (12). Composite hemangioendothelioma (CHE) is especially rare in clinical practice and is classified as an intermediate-grade tumor (15). CHE is composed of various histological components, including benign vascular tumors (such as cavernous hemangioma), intermediate-grade vascular tumors (such as retiform hemangioendothelioma), and malignant vascular tumors (such as epithelioid hemangioendothelioma) (13, 16). CHE is comprised of at least two or more of these components (17). The proportion of vascular components varies among individuals, with distinct ratios observed in different patients (18). CHE almost always expresses CD31, EGR, and Fli1, while over 50% of cases also exhibit CD34 and D2–40 expression (6). Most cases of CHE are found in young to middle-aged adults (11).

Currently, clinical treatment of CHE involves surgical resection, radiation therapy, and interferon therapy, primarily informed by case reports (10, 19). Complete surgical resection continues to be the main treatment modality for CHE and has been associated with excellent clinical outcomes. CHE exhibits local invasiveness but rarely metastasizes (20). The local recurrence of the tumor is closely associated with incomplete surgical resection. Additionally, the invasive nature of tumor cells and the high permeability of CHE may contribute to insufficient tumor removal (21). Apart from surgery, Lacin et al. reported a case of HE that was treated with radioembolization. After an 18-month follow-up, the patient had peritoneal involvement and even liver failure, eventually leading to death. The patient had received chemotherapy and interferon therapy prior to radioembolization, but the tumor continued to progress (12). According to the work of Sakamoto A et al, Patients with multiple tumors of the foot and sole underwent extensive resection and radiotherapy, and were followed for 2.5 years through 18F-FDG positron emission tomography, with no recurrence or metastasis detected. However, some patients do not respond to chemotherapy (14, 16). Radiotherapy can be employed as an adjuvant treatment following surgical procedures, and it has also demonstrated satisfactory outcomes in cases of post-surgical recurrence (14).

Although radical surgery combined with adjuvant precise radiotherapy has demonstrated excellent therapeutic outcomes in terms of local control rates and long-term survival (7, 9, 22), some patients are either unable to tolerate the procedure or cannot achieve complete resection. For these individuals, it is essential to explore alternative treatment options beyond surgical intervention. According to pertinent reports, only a limited number of studies have documented the use of radiotherapy as a curative treatment for CHE. Nakamura et al. reported a case of a patient with a cervical spine vascular endothelioma who underwent radiation therapy following incomplete resection of the tumor. After a 21-month follow-up period, no recurrence or metastasis was observed (7). In this case, given the complexity of the patient’s condition and the challenges associated with surgical intervention, we opted for radiotherapy as the first-line treatment. Following the completion of radiotherapy, a lumbar MRI revealed that the CHE lesions were effectively controlled, and the associated pain symptoms had resolved. These findings suggest that radiotherapy may serve as a viable alternative treatment for patients with unresectable tumors. In addition, the exploration of biological agents as alternative treatment options to surgery has also been undertaken. A case report described the treatment of a lesion located in the distal part of the left forearm and hand, where interferon alpha-2b was administered as the first-line therapy, resulting in significant recovery (10). This suggests that subcutaneous interferon therapy may represent a convenient and effective alternative.

## Conclusion

In conclusion, CHE is a rare borderline vascular neoplasm. Primary spinal CHE represents an even rarer clinical condition. According to relevant reports, complete surgical resection remains the standard treatment approach for CHE. Radiotherapy and interferon therapy may serve as viable alternative treatments for patients with unresectable tumors. However, given that all relevant reports are currently case reports, the level of evidence remains relatively low. Additionally, assessing patient prognosis necessitates longer-term follow-up and observation. Therefore, further exploration and case accumulation will be required in the future.

## Data Availability

The original contributions presented in the study are included in the article/supplementary material, further inquiries can be directed to the corresponding author.
